# Microbial Biocontainment Systems for Clinical, Agricultural, and Industrial Applications

**DOI:** 10.3389/fbioe.2022.830200

**Published:** 2022-02-02

**Authors:** Aaron Pantoja Angles, Alexander U. Valle-Pérez, Charlotte Hauser, Magdy M. Mahfouz

**Affiliations:** ^1^ Laboratory for Genome Engineering and Synthetic Biology, Division of Biological Sciences, King Abdullah University of Science and Technology (KAUST), Thuwal, Saudi Arabia; ^2^ Laboratory for Nanomedicine, Division of Biological Sciences, King Abdullah University of Science and Technology (KAUST), Thuwal, Saudi Arabia

**Keywords:** biocontainment systems, bioproduction, bacterial release, synthetic biology, drug delivery, microbial engineering

## Abstract

Many applications of synthetic biology require biological systems in engineered microbes to be delivered into diverse environments, such as for *in situ* bioremediation, biosensing, and applications in medicine and agriculture. To avoid harming the target system (whether that is a farm field or the human gut), such applications require microbial biocontainment systems (MBSs) that inhibit the proliferation of engineered microbes. In the past decade, diverse molecular strategies have been implemented to develop MBSs that tightly control the proliferation of engineered microbes; this has enabled medical, industrial, and agricultural applications in which biological processes can be executed *in situ*. The customization of MBSs also facilitate the integration of sensing modules for which different compounds can be produced and delivered upon changes in environmental conditions. These achievements have accelerated the generation of novel microbial systems capable of responding to external stimuli with limited interference from the environment. In this review, we provide an overview of the current approaches used for MBSs, with a specific focus on applications that have an immediate impact on multiple fields.

## Introduction

The field of synthetic biology aims to develop engineered organisms able to execute programmed tasks under a plethora of environmental conditions and scenarios. However, a significant concern is how these engineered organisms could affect the environment where they are released. For example, in environmental and agricultural applications, these organisms could affect surrounding species through the release of genetic circuits or engineered DNA and their potential incorporation into natural ecosystems (Berg et al., 1975; Bøhn et al., 2016; Lee et al., 2018). In medical applications, engineered microorganisms can efficiently colonize organs and tissues for bacterial therapies, however, this brings concerns about pharmacological control and the risk of transferring the genetically engineered material to the human microbiome (Mimee et al., 2016; Sarotra and Medhi, 2016). For this reason, several MBSs have been designed to control the proliferation of these organisms or prevent the environmental release of engineered genetic material (Curtiss et al., 1977; de Lorenzo, 2009).

**FIGURE 1:**
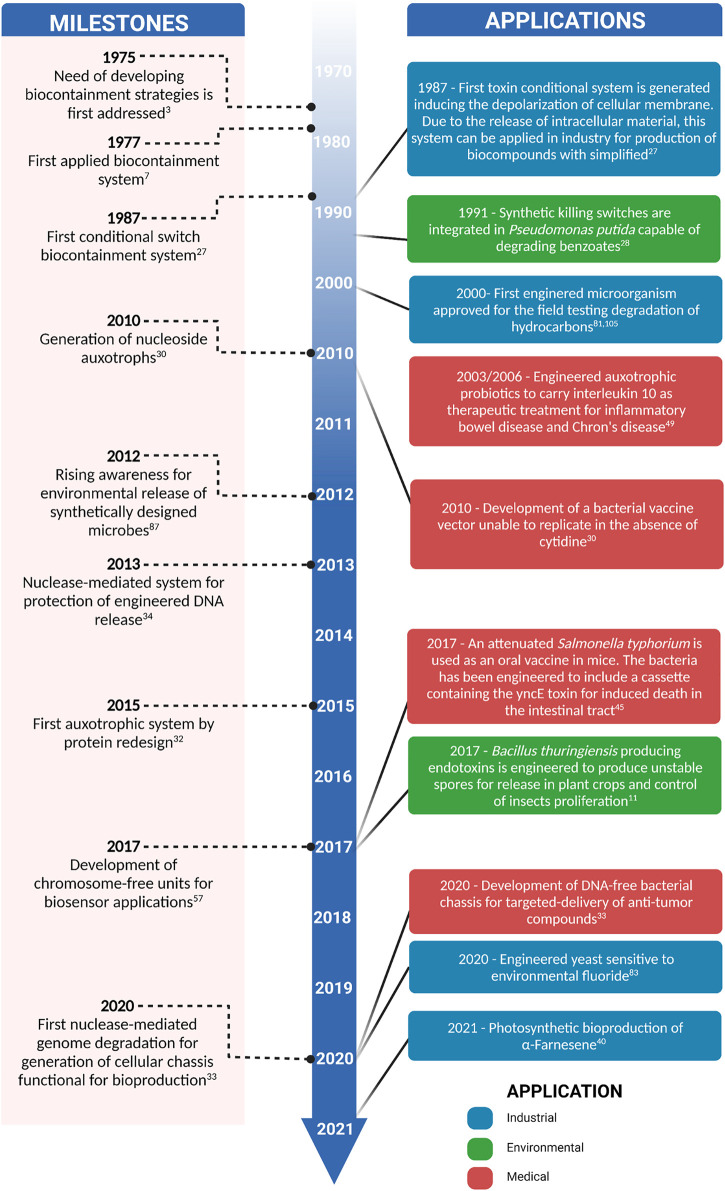
Timeline of milestones in the development of microbial biocontainment systems and important applications in the environmental, industrial, and health fields.

By taking advantage of the inherent functionality of MBSs, these organisms can be used to undertake simple or complex tasks, such as the production of proteins or the execution of metabolic pathways that lead to the biosynthesis of important secondary metabolites (Stargardt et al., 2020; Lalwani et al., 2021; Malla et al., 2021). Overall, MBSs have important agricultural, industrial, and medical applications (Figure 1). For example, MBSs have been used as vectors for toxins that associate with several types of crops for the efficient control of pests (Park et al., 2017). They have also been injected into tumors for the targeted biosynthesis and delivery of anti-cancer drugs (van Zandwijk et al., 2017). Moreover, MBSs have been used to simplify protein purification processes at the industrial scale, where they are programmed for induced autolysis to facilitate the release and purification of recombinant proteins (Morita et al., 2001). Thus, the development of novel approaches for the biocontainment of modified microorganisms has already yielded important progress, opening novel possibilities for future applications.

A major advantage of MBSs is their ability to execute *in situ* tasks without the risk of compromising the surrounding biological systems. Moreover, with the power of synthetic biology, these organisms can adopt genetic circuitries that allow environmental signals to trigger the execution of programmed tasks (Cubillos-Ruiz et al., 2021; McNerney et al., 2021). This dynamic responsiveness allows for applications that are condition-sensitive, in other words, programmed responses that are modulated based on environmental inputs (Chen et al., 2016; Yin et al., 2017; Zheng et al., 2019). The great potential of this promising concept has already been exploited to trigger apoptosis only in cells that have a malignant profile (Xie et al., 2011).

MBSs as biological factories have emerged as solid platforms for the *in situ* delivery of bio compounds. These robust systems have been utilized across different bacterial and yeast species and have been engineered to maintain tight regulation over the proliferation of engineered organisms while achieving high yields for easy scalability. In this review, we offer an overview of the most common MBS platforms, categorizing them by the cellular layers they act upon. We also highlight their most intriguing applications for the medical, environmental, and industrial fields and discuss emerging biotechnological applications for next-generation bioproduction.

## Overview of Microbial Biocontainment Systems

A microbial biocontainment system (MBS) is a platform used to limit microbial proliferation in systems that perform biological processes under controlled conditions. Various molecular strategies have been used to accomplish this goal, and mostly rely on human or environmental inputs to activate the inhibitory mechanisms. Some of the inputs are chemicals (like IPTG, arabinose, tetracycline) while others are changes in conditions (temperature, pH, UV light) that ultimately triggers the activation of designed genetic circuits that ultimately mediate the microbial growth inhibition. The ability to switch between the proliferative and inhibitory states is essential for maintaining a population of cells from which new MBSs can be generated; microbes in a controlled proliferative state are needed for generation of new batches that can be subsequently used in an inhibited state for any of the desired applications. Importantly, all mechanisms used to restrain microbial growth must interfere with one of the three fundamental cellular layers (protein, RNA, or DNA); by doing so, central processes in microbes are arrested and growth inhibition is achieved.

**FIGURE 2:**
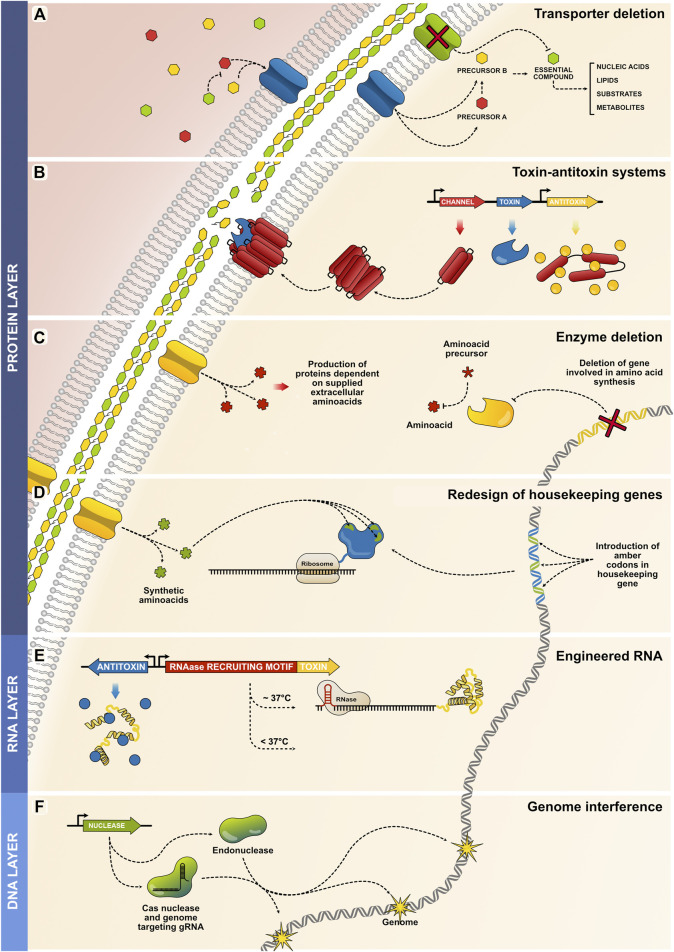
Strategies for modulating microbial propagation across the protein, RNA, and DNA layers. **(A)** Auxotrophy by transporter deletion is achieved by deleting a gene encoding a membrane transporter required to import an essential compound (green channel and green hexagon) required for maintaining central cellular processes. Thus, strict supplementation of precursors (red and yellow hexagons) is needed to ensure cell survival. **(B)** Toxin-antitoxin systems elicit cell death via various mechanisms. A common mechanism is the degradation of the bacterial cellular wall by catalytic enzymes (blue toxin), which compromises its structural integrity and elicits cellular bursting. Accessory elements such as membrane channels can be added to facilitate the activity of the toxin protein. Ultimately, the antitoxin is constitutively expressed to impede the activity of any toxic that might be expressed due to the innate leakiness of the promotor. **(C)** A housekeeping gene is recoded to incorporate amber codons that are used for the incorporation of NSAAs. The recoded sequence can produce a functional housekeeping protein (blue protein with green spots representing the positions of the incorporated NSAAs). Depleting extracellular NSAA concentrations leads to the improper translation of the essential protein, resulting in cell death. **(D)** In this example of auxotrophy by enzyme deletion, an amino acid-synthesizing enzyme is deleted. As a consequence, exogenous amino acids must be provided for the cell to sustain intracellular protein production. **(E)** Temperature-dependent translation of toxins can be regulated at the RNA level; the RNA is designed to contain an RNase-recruiting motif that acquires its functional state at a specific temperature range. Thus, upon changes in temperature, the structure is destabilized and the toxin expressed. **(F)** Genome interference is achieved by the overexpression of nucleases that target different sites of the genome, causing the intracellular exonucleases to digest the genome and compromise its integrity.

**Protein layer.** Most MBSs rely on protein-based mechanisms; the broad range of proteins allows different approaches to be designed for attaining biocontainment. Several strategies have been established involving the deletion of essential proteins (membranes, enzymes) or the expression of threatening ones (toxins), both approaches leading to cellular death. The first category of containment, auxotrophy by transporter deletion, is achieved by deleting a membrane transporter gene required to import an essential compound for cellular maintenance (Figure 2A). By impeding the importation of an essential compound, the microorganism is no longer able to sustain fundamental processes, and in consequence, die. Thus, precursors of the essential compound must be provided so they can be imported by other available transporters, this way, the cell can use the precursors to synthesize the essential compound and maintain viability (Hirota et al., 2017). Therefore, controlling the availability of the precursor is then the mean used to control the proliferation capability of the microorganism. Another category is the pioneering biocontainment system known as the toxin–antitoxin system. There are different modules and versions, but all rely on the integration of a synthetic circuit containing a gene coding for a toxin that interferes with bacterial viability. This gene is kept silent until desired and is stably controlled by an inducible promoter. To enhance the steadiness of the system, an antitoxin gene is also integrated under the control of a constitutive promoter (Contreras et al., 1991; Denkovskienė et al., 2020; Hayes and Kędzierska, 2014; Molin et al., 1987; Morita et al., 2001; Pasotti et al., 2011; Peng et al., 2019; Stirling et al., 2017; Zhou et al., 2019). The antitoxin prevents any leaked toxin from triggering cell death, and its suppression is only overcome upon the expression of the toxin (Figure 2B). Other methods depend on auxotrophy by enzyme deletion (Figure 2C). By removing enzymes involved in the synthesis of specific amino acids or metabolites, the cellular system becomes dependent on the extracellular sources from which they can obtain the essential compound. External availability is then needed for preserving cellular functions and lack of artificial supplementation restricts the viability of the biological system (Bahey-El-Din et al., 2010; Lindner et al., 2018; Steidler et al., 2003). A more sophisticated method relies on auxotrophy by redesign of house-keeping genes (Figure 2D); these systems rely on the selection of housekeeping proteins that are redesigned to incorporate artificial non-standard amino acids (NSAA) within their core. Expression of these redesigned housekeeping proteins become essential for maintaining the functions and viability of the cellular system of interest. However, because the expression of the redesigned housekeeping genes depend on the presence of NSAAs, extracellular restriction of NSAAs will lead to improper protein translation, resulting in the loss of function of the protein and cellular death (Mandell et al., 2015).

**RNA layer.** The control of microbial proliferation can be also modulated at the RNA level. For example, the successful expression of toxin proteins can be regulated at the transcript level by adding an RNAase recruiting domains to the toxin transcript (Figure 2E). Thus, successful recruitment of the RNAase leads to unsuccessful expression of the toxin and continuous cellular viability, However, by integrating a temperature-dependent RNAase recruiting domain in the toxin transcript, changes in temperature can regulate the successful loading of the RNAase and lead to the successful/unsuccessful degradation of the toxin transcript (Stirling et al., 2017).

**DNA layer.** Nuclease proteins can be used to fragment and degrade genomic regions, ultimately leading to cell death. While endonucleases can be used for bacteria, other nucleases such as CRISPR/Cas can easily be programmed to function across multiple organisms with even higher specificities (Fan et al., 2020; Caliando and Voigt, 2015) (Figure 2F). Moreover, other platforms such as minicells offer cellular chassis containing no genomes, which can be released into the environment without the risk of proliferation (Farley et al., 2016; Rang et al., 2018).

**Environmental conditions.** Environmental factors can be used to restrict the growth of microorganism. For instance, the engineering of probiotics sensitive to temperature or to a specific component in the environment. However, these strategies are still permeable to the production of mutants, and have been reported to exceed the suggested escaping frequency for therapeutical applications (Mejía-Pitta et al., 2021). Another factor is the biocontainment based on cellular population densities in which bacteria are able to survive when being encapsulated inside of swarmbot capsules (Huang et al., 2016). On the other hand, the presence and absence of signaling molecules within the environment has also been exploited, an example of this are the Deadman and Passcode killing switches which rely on environmental inputs to control circuit function (Chan et al., 2016). More recently, the engineering of microalgae that proliferates at specific CO_2_ concentrations has been reported (Lee et al., 2021).

## Medical Applications

**FIGURE 3:**
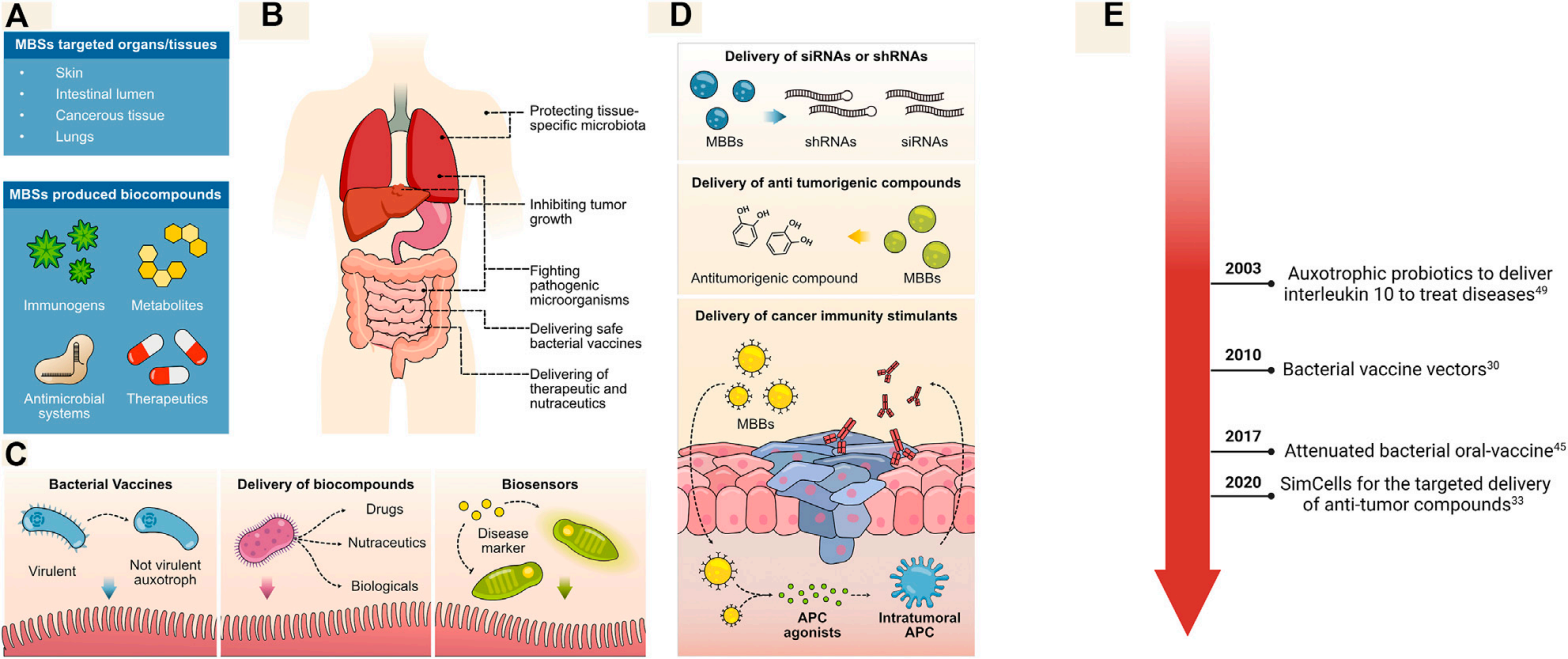
MBSs for clinical applications. **(A, B)** MBSs can be used to deliver a range of bio-compounds to different organs and tissues for diverse bacterial therapies. **(C)** The intestinal lumen is an attractive target for the delivery of non-replicative attenuated bacteria as vaccines (left-most panel, bio compounds (middle panel) and to test for the presence of diseases using available biomarkers (right-most panel). **(D)** Strategies used to treat tumorigenic tissues with MBSs, including the delivery of RNAs, drugs, and immune stimulants. MBBs are used for targeted delivery of shRNAs and siRNAs to tumors as a mean to knock down drug-resistance genes (top panel); MBBs can also be used as a direct biomanufacturing machinery for synthesis of antitumorigenic compounds (middle panel) or even for the delivery of agonists that will induce the development of an immunity response against the tumor (lower panel). **(E)** Timeline for major medical applications.

The limited proliferation of MBSs is a feature with important repercussions for the development of novel clinical techniques. As MBSs are metabolically active, they can be used as vectors for the *in situ* delivery of drugs and bio-compounds to the human body without the risk of invasion or sepsis (Figure 3A). This is possible because MBSs can be produced from microorganisms that naturally associate within specific human microbiota or prefer to develop in hypoxic conditions, such as tumor tissues (Kramer et al., 2018) (Figure 3B). This permits the generation of targeted therapies for cancer, treatments that beneficially alter the intestine or lung microbiota, the delivery of bacterial vaccines that can be removed upon the consumption of a specific sugar, and many other clinical applications (Ali et al., 2020). Furthermore, as MBSs can be used for the targeted delivery of drugs, lower doses could be administrated, potentially with the highest therapeutic effects, and reducing the probability of side effects (Odiba et al., 2016; Zhao et al., 2020).

A significant application is the delivery of MBSs to the intestines; this is an organ of interest as many therapeutics produced by MBSs are easily absorbed by the intestines. The intestine is an area of easy access, which only requires the ingestion of pills containing engineered bacteria. As an example, bacterial vaccines for intestinal delivery have been generated from attenuated strains; these are important tools for mass immunization, as they are easier and cheaper to produce than other types of vaccine. However, methods were needed to limit the continuous proliferation of the bacteria inside the intestinal lumen or after evacuation. As a mean to address these concerns, MBSs from attenuated *Salmonella typhimurium* have been designed to include a toxin system (initially tested in *Escherichia coli*) which is activated upon ingestion of arabinose (Figure 3C) (Li et al., 2016; Wang et al., 2017).

Furthermore, many human disorders arise from the dysregulation of the intestinal microbiota (such as Inflammatory Bowel disease) and could be treated by introducing designed bacteria (Oka and Sartor, 2020; Basso et al., 2019). These bacteria can deliver specific biologicals or act as sensors that recognize disease markers (Braat et al., 2006). However, a major concern comes upon evacuation of the bacteria as they might proliferate in the environment. Thus, MBSs can be used as a mean to detect temperature changes and interfere in the bacterial growth (Stirling et al., 2017). This can be accomplished by incorporating a system where a toxin is expressed by a temperature-sensitive RNA system (as described in Figure 2E). Thus, evacuation of the bacteria in lower-temperatures environments will interfere with its proliferative capabilities. This also opens up a range of possibilities for designing MBSs that contain sophisticated genetic circuits able to produce therapeutics only upon sensing specific conditions or disease biomarkers (Barra et al., 2020; Scott et al., 2021).

While many clinical applications can be developed for the delivery of MBSs to the intestines, others require direct interaction of MBSs with the target tissue. Malignant tumors develop unique metabolic environments for which novel therapeutic approaches can be designed to improve current treatments (Läsche et al., 2020; Sieow et al., 2021). Thus, certain bacterial strains can be used for targeted delivery of therapeutics, as they can naturally associate within hypoxic environment found in tumors or have been designed to stimulate the association (Figure 3D) (Zhao et al., 2005; Mandell et al., 2015; Duong et al., 2019). The non-pathogenic strain *Escherichia coli Nissle* was recently engineered to deliver Stimulator of Interferon Genes (STING) agonists directly to intra-tumoral antigen-presenting cells (APCs) (Leventhal et al., 2020). STING agonists mediate the generation of immunological memory, which has shown to restrict tumor colonization and affect tumor growth. The proliferation of the bacterial strain across other tissues was prevented by removing the thymidylate synthase (*thyA*) and tetrahydropicolinate synthase (*dapA*) genes, which are required for the production of thymidine and diaminopimelic acid. Thus, the restricted supplementation of thymidine and diaminopimelic acid into the tumor following bacterial implantation prevents the bacteria from proliferating (Figure 3D, bottom panel).

Another biosafe platform engineered for cancer therapeutics are minicells. These small, genome-free bacterial bodies are naturally produced from rod-shaped bacteria by aberrant divisions and can have been exploited for delivery of bio compounds (Farley et al., 2016). To increase the rate formation of minicells, *minD* or *minCDE* genes can be intentionally deleted in the parental cells; thus, improving the number of generated minicells for clinical applications (Rampley et al., 2017; MacDiarmid et al., 2009). These bodies have been specifically exploited for the targeted delivery of small interfering RNAs or short hairpin RNAs to knock down the Multidrug Resistant P-glycoprotein gene *MDR1* (Figure 3D, top panel). This system is further enhanced by inducing the expression of surface bispecific antibodies capable of recognizing and attaching to tumor-cell receptors (MacDiarmid et al., 2009). The direct administration of minicells containing siRNAs with the consecutive administration of minicells loaded with cytotoxic drugs effectively eliminated drug-resistant tumors. Along the same lines, other types of cancers such as malignant pleural mesothelioma tend to downregulate the microRNA miR-16, which acts as a tumor suppressor (Reid et al., 2013). Therefore, minicells loaded with miR-16 could be used to compensate for its downregulation and help repress tumor growth.

Minicells maintain intact transcriptional and translational machineries that can be used for expressing proteins coded in plasmids inherited from the parental cells. Thus, anti-tumorigenic compounds can be produced from plasmid-based genetic circuits once minicells have been delivered to the tumors (MacDiarmid et al., 2016; Zhang et al., 2018). In parallel, other genome-free platforms have been obtained by inducing the degradation of the native bacterial chromosome. This is done by inducing the expression of I-CeuI nuclease upon treatment with tetracycline (Fan et al., 2020). The bacterial chassis can be successfully used for the production of catechol, which was shown to interfere with the cellular viability of cancer cell lines (Figure 3D, middle panel). An important feature of these system is that they can be stored at low temperatures while preserving their functionality; this facilitates their development as clinical products that could be delivered and stored in hospitals until required (Fan et al., 2020).

Another attractive feature of chromosome-free MBSs is their programmability as biosensors; these systems have shown to be efficient at expressing fluorescent proteins as reporters in the presence of aspirin, glucarate, acrylate, or arabinose in proof-of-concept studies (Rampley et al., 2017; Chen et al., 2019). Therefore, minicells could be programmed to detect disease markers or metabolites present under specific health conditions (Figure 3C, right-most panel). A staggering number of possible combinations could be exploited to construct and program MBSs, representing an emerging field of study with promising applications.

## Industrial Applications

**FIGURE 4:**
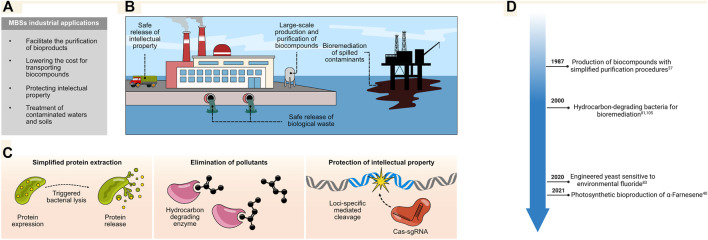
**(A,B)** MBSs can be used in various industrial processes ranging from the large-scale production of bio-compounds to the protection of sensitive material, the treatment of biological waste, and even the decontamination of accidentally released harmful material. **(C)** Different strategies have been designed to facilitate the purification of bio-compounds produced at a large scale; some of these rely on induced autonomous bacterial lysis (left panel). Other efforts are directed at the elimination of toxins released in the environment by integrating enzymes able to degrade complex hydrocarbons into bacteria; biosafety of the introduced genetic circuitries arises from their loss of function once the contaminants are depleted (middle panel). The protection of intellectual property can be achieved by inducing damage across the genome of the strain of interest (right panel). This can eliminate engineered genetic regions or even inhibit the proliferation of the MBS. **(D)** Timeline for major industrial applications.

Several biotechnological techniques with industrial applications rely on the production of enzymes, food derivatives, biofuels, and other biological compounds used as raw materials for the generation of products (Luan and Lu, 2018; Gilmour, 2019; Yushkova et al., 2019; Karim et al., 2020). Manufacturing these compounds from microorganisms requires methods for the efficient lysis of the microbe and purification of the desired bioproducts (Gao et al., 2013). Some MBSs allow for the autonomous disruption of the cellular membrane, enabling cost-effective extraction on an industrial scale (White et al., 2011). Other MBSs integrate circuits that elicit genome degradation, which enhances protein purification efforts in many processes where no DNA contamination is required (Caliando and Voigt, 2015). Furthermore, they can also be used for bioremediation purposes to eliminate contaminants from soils and sludges. Hence, MBSs can be used under different modalities to facilitate various industrial processes (Figure 4A, B).

One classical system for induced membrane disruption and the release of proteins was inspired by the lytic activity of T4 and T7 phage components (Smith et al., 1998; Duerkop et al., 2014). The holin and lysozyme enzymes from these phages provide the basis for a toxin system that can be integrated into bacteria under the control of inducible promoters. For instance, this system can be made sensitive to glucose starvation, the presence of IPTG, N-3-oxohexanoyl-l-homoserine lactone, or even heat (Morita et al., 2001; Yun et al., 2007; Pasotti et al., 2011). Once the expression of the circuit has been triggered, holin will accumulate and permeabilize the bacterial inner membrane, producing lesions and allowing the muralytic activity of the lysozymes to cleave and degrade the peptidoglycan layer (Zhang and Studier, 2004). The system was improved by adding an anti-holin into the toxin circuit under the control of a constitutive promoter; this antitoxin inhibits the activity of any leaked holin in the repressed state (White et al., 2011; van den Esker et al., 2017). Overall, this system can be successfully exploited across bacterial species to produce bio compounds on an industrial scale while circumventing the expensive traditional lysis methods (Borrero-de Acuña et al., 2017) (Figure 4C).

MBSs can also be used to treat industrial biological waste. Indeed, biosafe microorganisms have been metabolically engineered to help clean water and remove soil contaminants derived from industrial activity (Kour et al., 2021; Varjani et al., 2021). The intentional or accidental release of toxic compounds into the environment during industrial production is a major concern for which several bioremediation technologies are being developed (Verma and Sharma, 2017; Leong and Chang, 2020; Rylott and Bruce, 2020; Rahman and Singh, 2020). For example, hydrocarbon-consuming bacteria have been selected or designed to remove contaminants from sludges and sediments (Figure 4C) (Xu et al., 2018). However, the potential impact of these bacteria on the ecosystem following their release is a major concern. Nonetheless, some studies have shown that bacterial vectors can be used for the delivery of plasmids to the microbiota found in contaminated soil; this enhances the rate at which plasmid-encoded enzymes can digest petroleum hydrocarbons. Once the carbon source is removed, the plasmids disappear, most likely because they no longer provide a selective advantage (French et al., 2020). This represents a promising strategy for removing contaminants with minimal disturbance to the environment.

Another important application involves the protection of the intellectual property surrounding engineered microorganisms. Large amounts of funding and research efforts are needed to develop and design novel microorganisms that improve the yield and quality of industrial products, such as products derived from fermentation including wine, beer, and dairy products. One way to ensure that these strains are not used by third parties is to include a built-in biocontainment system for the autonomous removal of the engineered material. Using this technique, microorganisms that are released in the final product are not completely functional or no longer possess sensitive genetic information (Caliando and Voigt, 2015). As proof of concept, several systems have been shown to selectively remove genomic regions by harnessing the power of the CRISPR/Cas3 system where a gRNA is programmed to target the genomic region of interest (Caliando and Voigt, 2015). One convenient aspect of this platform is its programmability, as different gRNAs could be designed to remove virtually any sequence of interest (Figure 4C). This system could even be refined to induce irreversible genomic damage and inhibit the proliferation of the engineered strain.

Another major challenge is the disposal of biological cultures after their use. Traditional methods rely on costly and ecologically unfriendly methods such as chemical treatments, incineration, or autoclaving. Thus, MBSs that are sensitized to minerals in the environment could be used for biocontainment. For example, yeast cells lacking the fluoride exporter genes *Fex1/2* accumulate fluoride when present extracellularly. Thus, in controlled bioreactors lacking fluoride, the yeast would be able to proliferate and remain metabolically active; however, once released, environmental fluoride would accumulate intracellularly, eliminating the risk of propagation (Yoo et al., 2020). Since fluoride is naturally present in several types of environments, this mechanism offers an attractive alternative for designing industrial yeast strains for easy disposal.

## Agricultural Applications

**FIGURE 5:**
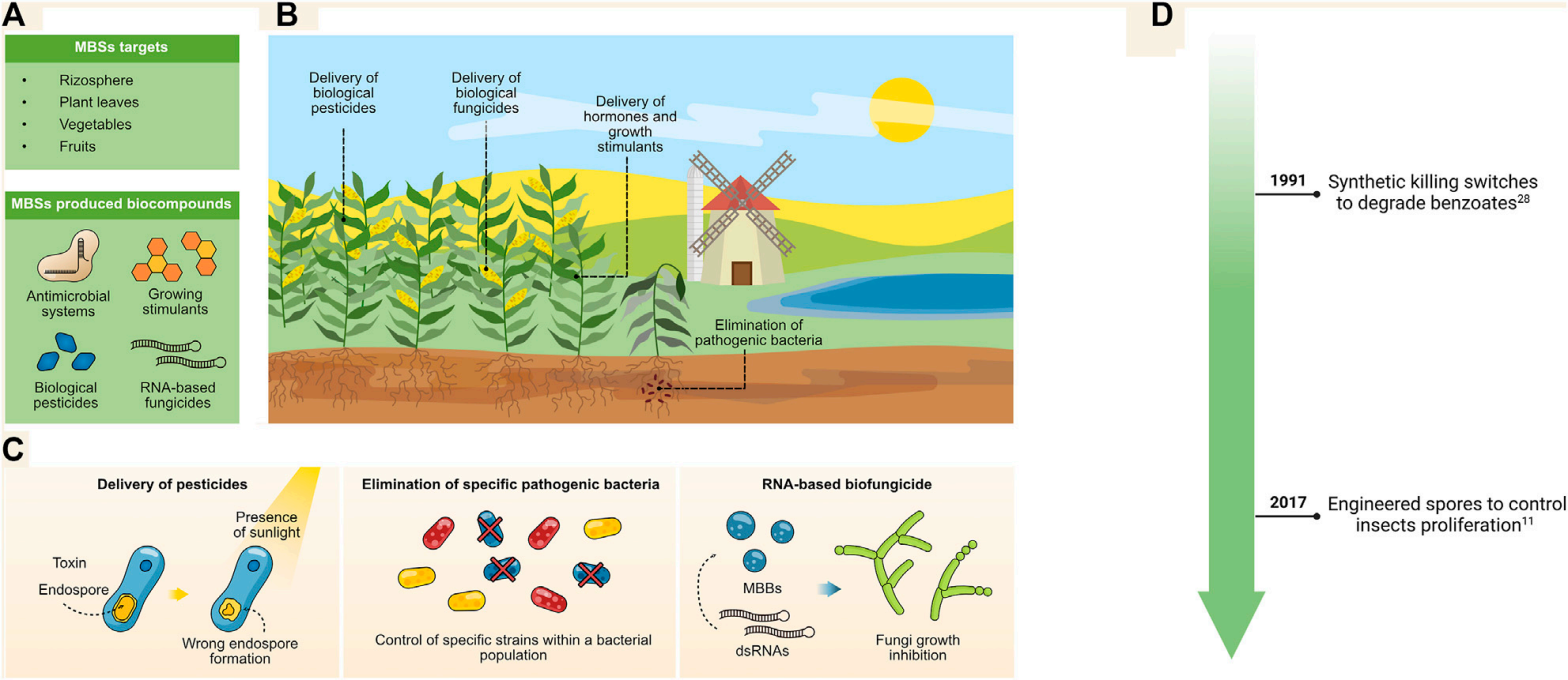
**(A,B)** MBSs can be used for the delivery of antimicrobial systems and biologicals in crop fields. **(C)** MBSs that produce toxins can be sensitized to UV, allowing them to deliver biological pesticides and be removed upon exposure to sunlight (left panel), they can also be engineered to deliver antimicrobial systems for the removal of pathogenic strains trough CRISPR/Cas-based genetic devices (middle panel) or by delivery of RNA-based knockdown devices. **(D)** Timeline for major environmental applications.

The agricultural field is one of the major activities required to sustain the constant food population demands. Constant biotechnological innovations are required for improving agricultural processes. Engineered microbes have shown to be important platforms for the delivery of pesticides, hormones, and growth stimulators to enhance crop yields (Backer et al., 2018; Ramakrishna et al., 2019; Glare and O'Callaghan, 2019). Moreover, bacteria can be used for cost-effective immunization processes needed in livestock to comply with international regulations. Nonetheless, the direct release into the environment of such designed microbes bring new concerns as they can easily propagate across fields and ecosystems (Dana et al., 2012). Thus, biocontainment measures are needed to inhibit their propagation across agricultural fields (Figure 5A, B); for this, various molecular strategies have been developed to accomplish these goals.

*Bacillus thuringiensis* is a highly attractive bacterium for agricultural purposes; its production of ∂-endotoxins makes it an ideal candidate for controlling insects in crop fields. However, the persistence and propagation of *B. thuringiensis* in the environment are a major concern to environmentalists. Park et al. removed the *spo0A* (sporulation master regulator) and α/ß-type Small Acid-soluble Spore Protein (SASP) genes from *B. thuringiensis*. The resulting strain produces unstable spores that are sensitive to UV exposure and sunlight (Figure 5C, left-most panel). Hence, the delivery of this bacterium in crop fields is strictly controlled by daylight, which interferes with the development of new spores for propagation (Park et al., 2017). In parallel, other organisms like *Agrobacterium tumefaciens* has been engineered to integrate a toxin-antitoxin system based on the inducible expression of PemK and PemI; while this system has been employed so far for the *in situ* engineering of plants, it could be used for the delivery of synthesized hormones and stimulants (Denkovskienė et al., 2020).

Another major problem in agriculture is the presence of undesired pathogens that associate with the leaves or rhizospheres of plants (Mendes et al., 2013). These pathogens are capable of damaging entire crop fields, prompting the need for new strategies for their control (Syed Ab Rahman et al., 2018). Novel technologies have been developed to deliver conjugation plasmids carrying a CRISPR/Cas system that will trigger cell death upon reception by the selected pathogen (Figure 5C, middle panel) (Hamilton et al., 2019). This system can be expanded to integrate a gRNA that will trigger the death of the bacterial vector, thereby removing any traces from the CRISPR/Cas system. As this is a highly programmable system, nearly any bacterial pathogen able to be conjugated could become a target. Furthermore, targeting specific microbes within a population can be enhanced by selecting pathogen genomic regions that are not shared among other microbes; thus, reducing the possibility of interfering with other microbial strains contained in the population.

Additionally, dsRNAs has demonstrated to be a potential technology for fungi control in crops; they can be processed into small interference RNAs (siRNAs) to mediate the growth inhibition of mold (Islam and Sherif, 2020). However, an important constrain is the stability of these RNAse in the environment for which using protective biological chassis is a mean to improve its stability. Minicells have proved to be stable non-replicative carriers of dsRNAs (Figure 4C, right-most panel); indeed, they have been engineered to compromise the activity of endogenous RNAase III, making them suitable as RNAs vectors (Islam et al., 2021). These systems have been *in vitro* delivered to strawberries and shown to inhibit growth of *Botryotinia fuckeliana* fungi by knocking down chitin synthase class III and DICER-like proteins. This system demonstrates the potential of MBSs for novel RNA-based bio fungicides.

## Future Applications

The ongoing development of enhanced and safer systems for bioproduction opens up the possibility of using engineered biological systems in environments that require higher levels of biosafety (Whitford et al., 2018). Some of these pioneering applications include bacterial therapy for personalized human health (drug delivery, biosensors, bacterial vaccines) and emerging areas such as astropharmacy (space bioproduction). It’s important to highlight that emerging biocontainment strategies aren’t restricted to be applied in prokaryotic systems. For instance, ion-inducible promoters, CO_2_ dependent, and toxin-antitoxin modules have been successfully engineered in eukaryotic microorganisms like microalgae (Young and Purton, 2016; Zhou et al., 2019; Lee et al., 2021). These advances show promising alternatives towards the bio-safer large-scale bioproduction.

There has been growing interest in developing novel platforms composed of programmable cells that can sense, target, deliver, and respond to different biological compounds for various therapeutic applications (Ates et al., 2020; Fritsche et al., 2020). Compared to previous approaches for drug delivery, whole-cell-based systems provide several advantages, including a robust chassis with increased specificity for the release of therapeutic compounds (Lin and Tao, 2017; Krinsky et al., 2018; Fan et al., 2020). For instance, minicells have been successfully used to inhibit tumor growth in patients, and SimCells are an emerging enhanced chassis with similar applications (van Zandwijk et al., 2017; Fan et al., 2020). Therefore, the on-site bioproduction of therapeutic compounds using safer bioproduction systems represents a novel approach with promising biomedical applications.

SimCells bioproduction systems have also been successfully engineered to act as biosensors (Chen et al., 2019), thereby contributing to the development of smart probiotics (Rottinghaus et al., 2020). Smart probiotics systems could be engineered to detect and release compounds within the human body without compromising human health, perhaps representing an important milestone for next-generation healthcare. The manufacturing of genetically modified T cells for therapeutic use has also been reported (Kaiser et al., 2015). Nevertheless, the on-site applications of cellular sensing systems are not restricted to point-of-care testing for clinical diagnosis, as they can also be used for environmental, industrial, and food home safety testing (Guo et al., 2021). One example is the engineering of biocontainment systems for genetically modified organisms (GMOs) for industrial purposes (Lee and Kim, 2015; Yoo et al., 2020). Novel biocontainment approaches for the continuous use of GMOs that can sense compounds without interacting with the external environment have also been introduced (Tang et al., 2021). Furthermore, the integration of stronger biocontainment systems contributes to the regulation of GMOs for industrial-scale applications and biorremediation (Ripp et al., 2000). The relevance of this is highlighted by the increasing number of regulations for the management of GMOs for larger-scale bioproduction. Hence, the increased biosafety within biocontainment systems contributes to the revolution in conventional bioproduction for the industrial and healthcare sectors.

Finally, apart from current biotechnology, the continuous development of space travel provides an unprecedented opportunity for the development of new bioproduction systems. A recent example was presented at the 2019 International Genetically Engineered Machine (iGEM) competition in the project “Towards an astropharmacy” (Hung, 2020). In general, astropharmacy centers on the production of pharmaceutical compounds during space travel, providing an alternative to contemporary challenges to space pharmaceuticals such as time degradation or irradiation, with the aim of enhancing the quality of astronaut healthcare. Recent experiments have been performed using cell-based and cell-free systems; however, incorporating novel bioproduction systems could contribute to a more robust, biosafe, long-term functional chassis with applications for space travel, such as the bioproduction of pharmaceutical compounds or even biosensors for healthcare monitoring in space. This highlights the potential for safer biocontainment systems as novel bioproduction platforms with future applications for biotechnology and healthcare.

## Concluding Remarks

Microbial biocontainment systems are platforms that have been refined in the past decade primarily to improve the inhibition of cellular proliferation. As a proof of concept, a range of applications involving the environmental release of MBSs have been developed. New strategies for improving important processes in the agriculture and healthcare industries could be developed based on these systems.
